# Synthesis of Chitosan-Based Bio-Polyols Using Glycerol and PEG-400 for Sustainable Semi-Rigid Polyurethane Foams

**DOI:** 10.3390/polym18212770

**Published:** 2026-11-06

**Authors:** Royal Guliyev, Nalan Tekin, Mustafa Özgür Bora, Yavuz Emre Yağci, Aygun Isayeva

**Affiliations:** 1 Department of Chemistry, Faculty of Arts and Sciences, Kocaeli University, Kocaeli 41001, Türkiye; 206132006@kocaeli.edu.tr; 2 Department of Airframe and Powerplant Maintenance, Faculty of Aeronautics and Astronautics, Kocaeli University, Kocaeli 41001, Türkiye; ozgur.bora@kocaeli.edu.tr; 3 Farplas Otomotiv A.S., Taysad Organize Sanayi Bölgesi (TOSB), Kocaeli 41420, Türkiye; e.yagci@farplas.com; 4 Department of Biomedical Engineering, Faculty of Technology, Kocaeli University, Kocaeli 41001, Türkiye; 206162004@kocaeli.edu.tr

**Keywords:** bio-based polyol, Chitosan-derived polyol, semi-rigid polyurethane foam, thermal stability, sustainable polyurethane

## Abstract

Replacing petroleum-based polyols with renewable feedstocks is essential for sustainable polyurethane (PU) production. In this study, an eco-friendly bio-polyol was synthesized using chitosan, glycerine, and PEG-400 to prepare semi-rigid bio-based PU foams at 50%, 75%, and 100% substitution ratios. The chemical, morphological, mechanical, and thermal properties were systematically evaluated. FTIR confirmed successful bio-polyol integration, while SEM revealed refined cell-dimensions and enhanced structural homogeneity. Although the apparent density decreased from 0.0835 g/cm^3^ to 0.0657 g/cm^3^, the bio-based foams exhibited superior water resistance and mechanical performance with the 75% substituted formulation (75 Bio PU). To justify field performance under demanding operational environments (e.g., building envelopes under solar heat load and hot pipeline insulation), the thermal conductivity was evaluated at 100 °C, maintaining efficient insulation (0.044–0.047 W m^−1^ K^−1^). TGA/DTG analysis demonstrated that while bio-polyol incorporation lowered the initial decomposition temperatures, 75 Bio PU enhanced the thermal resistance (T %max = 408 °C) and char residue (15.68%), underscoring superior high-temperature carbonization. UL-94 tests indicated the need for supplementary flame-retardants. Overall, the 75 Bio PU formulation provides optimal balanced performance for sustainable building insulation, industrial heat shielding, and automotive interior components.

## 1. Introduction

Polyurethane (PU) foams hold a significant position among polymeric materials with a wide range of applications [1,2,3]. The global polyurethane foam market is projected to reach approximately $54.3 billion by 2026 and to grow at a compound annual growth rate (CAGR) of 8.3% during the 2026–2033 period, reaching approximately $95.2 billion by 2033 [4]. This clearly demonstrates the rapid increase in the demand for PU foams.

Polyols (polyethers and polyesters) and isocyanates are the basic components of PU foam production [2,3]. However, as the primary raw materials for these chemicals are petroleum-derived, rising oil prices are causing price fluctuations in the global market. Furthermore, the high dependence on fossil-based components also raises serious environmental issues [5,6]. To mitigate the effects of these issues and develop more sustainable production methods, researchers have proposed various approaches aimed at utilizing renewable and bio-based raw materials [7]. In this context, studies on bio-based polyols and environmentally friendly additives hold significant potential for both reducing dependence on fossil resources and ensuring environmental sustainability [8].

The synthesis of bio-based polyols using sustainable resources involves the use of various biological feedstocks. In the literature, it is observed that within the scope of such studies, various vegetable oils [9], different types of starch, polysaccharides [10,11], and components such as lignin and cellulose have been evaluated as alternative feedstocks in polyurethane production [12,13]. These bio-based components are suitable for polyol synthesis because of the hydroxyl groups present in their chemical structures and their contribution to the development of environmentally friendly production processes [14].

Numerous studies have been conducted on the bio-based polyols derived from vegetable oils. In this context, various vegetable oils, such as castor oil, palm oil, canola oil, sunflower oil, flaxseed oil [15,16], soybean oil [17], waste oils, and olive oil, are converted into bio-based polyols through methods such as epoxidation, oxidation, and transesterification-based polyols and are utilized as raw materials in polyurethane foam production [18,19,20,21,22]. These bio-based raw materials have garnered attention because of their sustainability, eco-friendliness, and low carbon emissions. However, the fact that a significant portion of these raw materials has potential for use in the food industry or higher value-added sectors, coupled with structural limitations such as low reactivity and insufficient hydroxyl values, necessitates additional chemical modification processes. This situation limits the long-term cost-performance competitiveness of these raw materials and poses a serious obstacle to their becoming attractive alternatives on an industrial scale [23,24].

Consequently, approaches relying solely on vegetable oils will prove insufficient for solving all of these problems. In this context, there remains a significant need for research aimed at evaluating other bio-based resources, which are abundant in nature, as alternative raw materials.

Chitin is the most abundant amino-polysaccharide in nature. Although it is commonly found in the cell walls of crustaceans, insects, and fungi, its primary natural sources are shrimp and crab shells, from which approximately 6 million metric tons are harvested annually from coastal and inland waters [25]. Despite the abundance of natural chitin sources, industrial-scale applications remain limited because these byproducts are mostly treated as waste products and are not recycled. However, chitosan, an amino polysaccharide derivative obtained through the deacetylation of chitin, is widely used in numerous medical and biotechnological applications because of its advantages in terms of biocompatibility, biodegradability, and functional groups [26,27]. Recent studies have demonstrated that chitosan can be utilized as an additive [28] and crosslinking agent [29,30] in the polyurethane industry. These findings indicate that chitosan is a potential raw material for biomedical applications and the production of sustainable materials. However, owing to processing difficulties and poor solubility in its pure form, chitosan has traditionally been restricted to being used only as a filler or additive at very low concentrations to improve the mechanical and thermal properties of polyurethane foams modestly. To overcome these limitations, researchers have recently focused on liquefaction processes to synthesize liquid bio-based polyols directly from chitosan, thereby opening new pathways for its higher-value incorporation into polyurethane formulations [28,29,30]. Strzałka et al. (2023) investigated the use of chitosan in PU foams by modifying chitosan with different molecular weights using glycidol (GL) and ethylene carbonate (EC) via hydroxyalkylation and synthesizing bio-polyols for use in the production of bio-based polyurethane foams from chitosan [31,32,33]. This study clearly demonstrates the production potential of chitosan-based PU foams. However, considering the potential adverse health effects of components such as EC [34] and GL [35], as well as the cost-performance balance [36,37,38], further research is needed in this field using safer and more economically advantageous methods.

This study aimed to produce PU foam from sustainable sources using less harmful and more economical methods. To this end, a bio-based polyol was obtained by liquefying chitosan using glycerin and polyethylene glycol (PEG 400), and the goal was to evaluate these polyols as alternatives to fossil-based polyols in the production of semi-rigid PU foams. The bio-polyol obtained in this study was used to produce semi-rigid PU foams at different substitution ratios (50, 75, and 100%), and the thermal stability, mechanical performance, combustion behavior, morphological properties, water absorption capacity, and chemical structures of the produced foams were characterized. Thus, this study demonstrates the potential of chitosan-based biopolyols as sustainable, economical, and environmentally friendly alternatives for producing semi-rigid PU foams.

## 2. Materials and Methods

### 2.1. Materials

A three-functional polyether polyol (Mn ≈ 3000 g/mol; OH number 57; acid number ≈ 0.1; density 1.02 g/cm^3^ and viscosity 1570 mPa.s at 25 °C) and polymeric methylene diphenyl diisocyanate (PMDI; Lupranat^®^ M20S; density = 1.21 g cm^−3^, NCO content ≈ 31.5%, functionality ≈ 2.8, and viscosity = 155 mPa·s) were supplied by Farplas Otomotiv A.Ş. (Çayırova, Kocaeli, Türkiye). Chitosan, possessing a molecular weight between 20 and 350 kDa and a deacetylation degree of 90%, was obtained from Biyopol Company (Avcılar, İstanbul, Türkiye). Dibutyl tin dilaurate (DBTDL) catalyst, PEG 400 (99%), and Tegostab B 8404 surfactant were supplied by Kimpur (İstanbul, Turkey). Glycerin (99.9%), sulfuric acid (H_2_SO_4_, 99.8%), and sodium bicarbonate (NaHCO_3_, 99.9%) were obtained from Sigma-Aldrich (St. Louis, MO, USA).

### 2.2. Preparation of Liquid Chitosan

**Figure 1:**
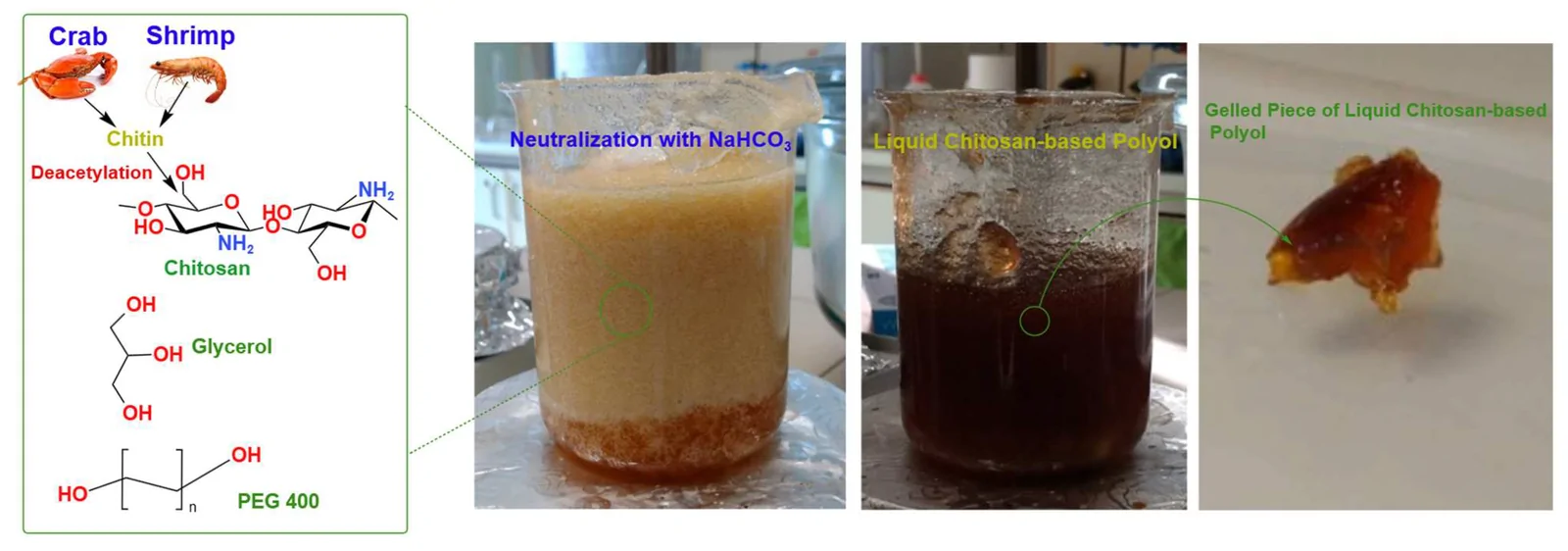
Schematic illustration of the synthesis process of chitosan derived bio-polyol.

The experimental procedure for the liquefaction of chitosan is described in detail below. First, 150 mL of distilled water containing 1% (*w*/*w*) acetic acid was prepared in a 1-L glass beaker. Ten grams of chitosan was added to this solution, and the mixture was stirred for 3 h at room temperature using a magnetic stirrer. During this process, a large portion of the chitosan was protonated and dissolved in the aqueous phase, resulting in a high-viscosity, gel-like, and homogeneous chitosan solution. Subsequently, 100 mL of glycerin and 50 mL of polyethylene glycol (PEG 400) were added to the reaction mixture, which was stirred at 350 rpm at room temperature for 1 h to ensure system homogeneity. To accelerate the liquefaction reaction, a catalytic amount (0.3% *w*/*w*) of sulfuric acid was added to the system. The mixture was then heated to 85 °C and allowed to react for 3 h while maintaining the same stirring speed. To reduce the water content in the reaction mixture and increase the degree of liquefaction, the temperature was raised to 120 °C, and the system was stirred under these conditions for approximately 4 h. Upon completion of this stage, the temperature was lowered to 75 °C, and 2 g of sodium bicarbonate was added in a controlled and gradual manner to neutralize the sulfuric acid in the system. The overall mass yield of the resulting bio-polyol reached 91.24% of the initial biomass feed. The observed 8.76% mass reduction is primarily driven by the thermal volatilization of reaction by-products, most notably water liberated through condensation steps during acid-catalysed liquefaction. Following cooling, a dark-orange gel-like product was obtained (Figure 1). Following cooling, a dark-orange gel-like product was obtained (Figure 1).

### 2.3. Preparation of Polyurethane Foam

Bio-based polyurethane foams were prepared using a method similar to the conventional open-cell PU foam production method. For the polyol composition used in the foam formulations, 1% (*w*/*w*) water was added as a blowing agent, 1% (*w*/*w*) dibutyltin dilaurate (DBTDL) as a catalyst, and 0.5% (*w*/*w*) additive as a surfactant (Tegostab B 8404). These components were mechanically mixed at a rotation speed of 1000 rpm for 30 s to obtain a homogeneous premix.

**Figure 2:**
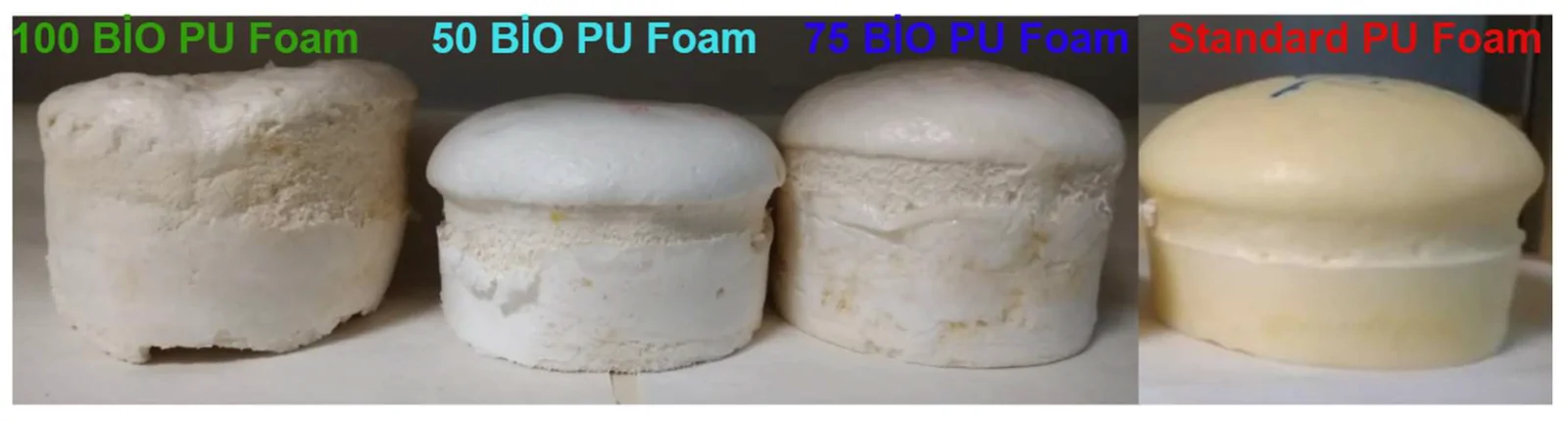
Images of standard PU foam and bio-based polyurethane foam samples made from CH/GL/PEG-400.

Considering the possibility of trace amounts of water remaining in the structure during the synthesis of the chitosan-based polyol, the amount of externally added water was reduced proportionally as the proportion of chitosan-based polyol in the formulation increased. This approach allowed for the control of the foaming reaction, thereby minimizing any adverse effects on the cellular structure. In this context, 50% of the total polyol amount was replaced with a chitosan-based polyol in the preparation of 50 BIO-PU samples. In the 75 BIO-PU samples, this ratio increased to 75%, and in the 100 BIO-PU samples, the entire polyol component (100%) consisted of chitosan-based polyols. All foam samples were prepared by mixing in a 550 mL circular polyethylene container at a rotation speed of 2200 rpm for 30–40 s. The detailed formulation information for the 50 BIO-PU, 75 BIO-PU, and 100 BIO-PU samples (Figure 2), along with that for the standard PU foam produced for comparison, is presented in Table 1.

In addition, the characteristic parameters related to the production process of the standard PU and bio-based PU foam samples, such as cream-up time, onset of foaming, free rise time, gelation time, and the NCO/OH index (calculated by incorporating water equivalents based on the formulations given in Table 1), are summarized in Table 2.

Upon examining the results, it was observed that the cream-up time, free rise time, and gelation time values of the PU foam samples produced using bio-based polyol were generally similar to those of standard PU foam, and that there were no significant differences in the process parameters. However, as the amount of bio-based polyol increased, a noticeable prolongation was observed, particularly in creaming time. This is attributed to the high viscosity and gel-like structure of the bio-based polyol. High viscosity slows down the homogenization of the mixture and the initial reactions between the isocyanates and hydroxyl groups, resulting in a longer whipping time.

The color characteristics of the synthesized foams revealed two distinct phenomena. First, despite the dark orange appearance of the synthesized liquid bio-polyol resulting from thermal modifications during chitosan processing, the resulting bio-foams exhibited a light/white color. This phenomenon was driven by intense multiple light scattering at the gas–solid cell wall interfaces within the microcellular structure, and this effect optically masked the natural color of the raw material. Second, foams containing bio-polyol demonstrated superior color stability over time compared to standard PU foams, which undergo noticeable yellowing over time. In traditional aromatic PU matrices, yellowing is typically caused by unreacted residual isocyanate (NCO) groups and subsequent formation of photooxidative quinone chromophores. The incorporation of a highly reactive bio-polyol into the structure promotes more efficient NCO consumption and increases cross-link density, thereby suppressing color change/yellowing in the foam by inhibiting oxidative degradation pathways.

This phenomenon was also evident in the FTIR analysis results for the PU foams (as confirmed by the reduction or disappearance of the NCO peaks).

### 2.4. Characterization of Bio-Polyol

A matrix-assisted laser desorption/ionization time-of-flight (MALDI-TOF) mass spectrometer manufactured by Bruker Daltonics (Bremen, Germany) was used to determine the molecular weight distribution of the bio-based polyol synthesized in this study. The analyses were performed in positive ion and linear modes. Measurements were conducted over a wide mass range (0–500,000 Da) to enable the detection of high molecular weight species. Dithranol (DIT) was used as the matrix during sample preparation, and the matrix solution was prepared using a mixture of 30% acetonitrile (ACN) and 70% water containing 0.1% *w*/*w* trifluoroacetic acid (TFA). The sample and matrix mixture were applied to the target plate using the “dried droplet” method. This method ensures homogeneous crystallization of the sample, thereby enhancing ionization efficiency.

The hydroxyl (OH) and acid numbers of the prepared bio-based polyol were determined in accordance with the ASTM D4274-11 [39] and ASTM D4662-08 [40] standards. The OH number was measured using a pyridine- and imidazole-based reaction system [41]. To evaluate the reproducibility of the analyses, two parallel samples, each consisting of three replicates, were analyzed. The imidazole-based method provided lower toxicity, safer working conditions, and higher measurement consistency than the pyridine-based method [42]. Therefore, all OH values reported in this study were strictly derived using the imidazole-based titration procedure. Titration was performed using a 0.05 M KOH/methanol solution with phenolphthalein as the indicator.

To investigate the structural characterization of chitosan-based bio polyol samples, FTIR spectra were obtained using Diamond ATR on a Bruker Tensor 27 spectrophotometer (Bruker Optics GmbH & Co. KG, Ettlingen, Germany) at 4000 to 400 cm^−1^ with 30 scans and 2 cm^−1^ resolution.

### 2.5. Characterization of PU Foams

A 72-h water absorption test was conducted to assess the water-absorption behaviour of the standard PU and CH/PU foam samples. The test was performed at room temperature to simulate standard conditions and prevent temperature-induced hydrolytic degradation or swelling. Five replicates were prepared for each formulation. Samples (approximately 30 mm × 30 mm × 20 mm) were dried in an oven at 75 °C for 24 h, and their initial masses (M1) were recorded. The samples were fully submerged in water. To ensure uniform surface contact and prevent movement, a glass plate of specific mass was placed on top and secured using its thin edge. After 72 h, the samples were removed, the surface water was gently wiped off with paper towels, and final masses (M2) were recorded. Water absorption capacities were calculated using Equation (1) [43,44]:

(1) $$\mathrm{Water}\mathrm{absorption}\mathrm{rate}\;(\%)=\frac{\left(M2-M1\right)}{M1}\cdot 100$$

*M*1: The initial dry mass of the sample,

*M*2: The mass measured after immersion in water.

The thermal behavior of standard SrPU, 25 BioPU, 50BioPU, and 75BioPU foam samples was determined by thermal gravimetric analysis (TGA; Mettler Toledo TGA 1, Mettler-Toledo International Inc., Greifensee, Switzerland) at a heating rate of 10 °C/min from 30 °C to 600 °C under an N_2_ atmosphere.

The compression samples were 25 mm × 25 mm × 25 mm in size, and the compression tests were conducted. Electronic universal testing machine (CMT4304-QY, MTS Systems Corporation, Eden Prairie, MN, USA) was used for quasi-static compression tests with a strain rate of 0.001 s^−1^, and the tests ended at 70% strain. Each compression experiment was repeated five times; the average values were taken as the experimental results. The compression modulus (E) was calculated as the slope of the steepest linear portion of the initial region of the stress–strain curve (within 0–8% strain). Because no stress maximum occurred before 10% relative deformation, the compressive strength was taken as the compressive stress at 10% relative deformation (σ_10_) in accordance with ISO 844; the compressive stress at 50% strain (σ_50_) and at the end of the test (70% strain, σ_70_) were also recorded. The collapse point (ε_c_, σ_c_) was determined from the intersection of the tangents to the linear-elastic and plateau regions. All parameters were evaluated on the average stress–strain curve of the five replicates for each formulation.

The thermal conductivity properties of the samples were assessed in accordance with the ISO 22007-2 [45] standard. Measurements were conducted under constant environmental conditions at a fixed temperature of 100 °C. All samples were prepared with dimensions of 50 mm × 50 mm × 5 mm (width × length × thickness) to fit the measurement device. Thermal conductivity analyses were performed in triplicate to enhance statistical reliability.

The apparent densities of the foam samples were determined based on ASTM D7710-14 [46], Standard Test Method for Determining Volume and Density of Rigid and Irregularly Shaped Molded Cellular Materials. The samples were prepared with dimensions of 30 mm × 30 mm × 20 mm (length × width × thickness). The volumes of the samples were determined using the water displacement method in a cylindrical container. After filling the container with water to a certain level, the sample was carefully immersed, and the difference in the water level was recorded as the volume of the sample. Each measurement was performed in a triplicate, and the mass was measured using an electronic balance. The apparent density of each sample was calculated by taking the average of the obtained mass–volume ratios, and the results were expressed in g/cm^3^. In addition, the average density and mean square deviation values obtained from the measurements were noted.

To obtain information about the surface of the Standard SrPU, 25 BioPU, 50BioPU, and 75BioPU foam samples, analysis was performed using a JEOL JSM-6060 scanning electron microscope (SEM) (JEOL Ltd., Akishima, Tokyo, Japan). The SEM was silver-coated and operated with a secondary electron detector to obtain detailed images of the surface morphology. Additionally, quantitative image analysis was performed on representative micrographs (at 25× magnification) using ImageJ (version 1.53n, National Institutes of Health, Bethesda, MD, USA) software to determine pore characteristics. The total number of pores and the fraction of cells exhibiting well-defined wall perforations and interconnected windows were systematically counted across multiple fields to evaluate the open-cell content percentage.

## 3. Results and Discussion

### 3.1. Characterization of Bio-Based Polyol

The acid and hydroxyl (OH) numbers of the synthesized bio-based polyol were determined by titrimetric analysis using a 0.05 M KOH/methanol solution. Phenolphthalein was used as an indicator in the titration, and the acid and hydroxyl numbers were calculated based on the volume of KOH solution consumed during the titration [47]. According to the analysis results, the acid number of the synthesized bio-based polyol was 4.1 mg KOH/g, and the hydroxyl number was 87 mg KOH/g. Although a low acid value indicates that the amount of free carboxylic acid remaining in the system is low, this low acid number should not be interpreted as direct and definitive evidence of complete esterification, given the use of a sulfuric acid catalyst during synthesis and the subsequent neutralization step with sodium bicarbonate. Instead, the success of the chemical modification was confirmed through FTIR and MALDI-TOF mass spectrometry analyses. Furthermore, the determined hydroxyl number (87 mg KOH/g) demonstrated that the polyol possesses sufficient hydroxyl functionality to react with isocyanates. Although the hydroxyl numbers reported in the literature for bio-based polyols vary significantly depending on the raw materials and synthesis methods used [48], the value obtained in this study falls within that range; this confirms that the synthesized polyol possesses the appropriate reactivity for polyurethane foam production.

### 3.2. MALDI-TOF MS

**Figure 3:**
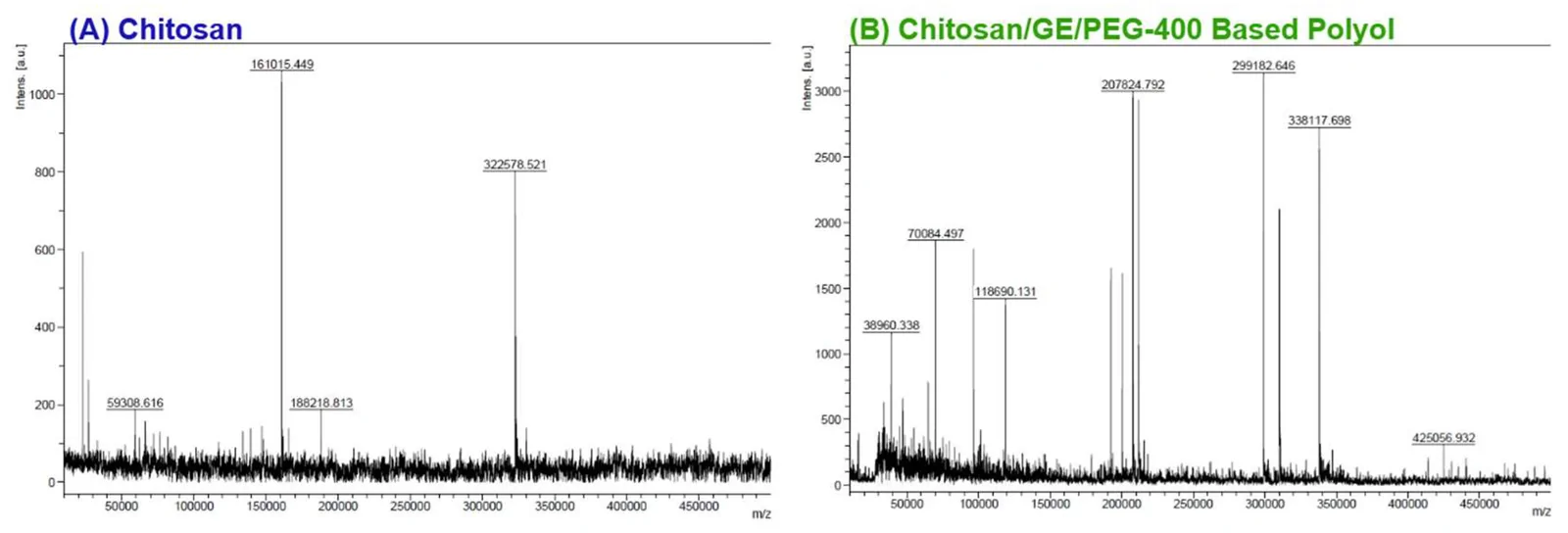
(**A**) MALDI-TOF MS spectra of chitosan and (**B**) bio-based polyol samples based on CH/GL/PEG-400.

MALDI-TOF MS was used to determine the molecular weight distribution, number-average molecular weight (Mn), and polydispersity index of the bio-based polyol obtained [33,34]. Because a significant portion of the synthesized polyol consisted of chitosan derivatives, MALDI-TOF MS analyses were performed on both pure chitosan and the bio-based polyol obtained using PEG-400, glycerol, and chitosan for comparative evaluation. The analysis results for chitosan are presented in Figure 3A and Table 3.

When the data from the figures and tables are evaluated together, it can be seen that the molecular weight distribution of pure chitosan exhibits a distinctly multimodal character. According to the MALDI-TOF MS results, chitosan was found to cluster into three distinct mass ranges: the low molecular weight region in the 23–66 kDa range, the medium molecular weight region in the 147–188 kDa range, and the high molecular weight fraction concentrated in the approximately 310–340 kDa range. In this distribution, the 147–188 kDa range was particularly dominant, with peak intensities and signal strengths reaching their maximum in this region.

As shown in Table 3, the molecular weight distribution clusters in three main regions: 23–66 kDa, 147–188 kDa (dominant distribution), and approximately 322 kDa. Because it is difficult to directly determine the Mn and Mw values in such heterogeneous systems, the average values were calculated based on the literature, considering these distribution regions.

In contrast, the molecular weight distribution of the bio-based polyol sample obtained by modifying chitosan spanned a wider range and shifted distinctly toward higher molecular weight regions (Table S1). The data obtained indicate that the concentration in the bio-based polyol increases in the regions of approximately 190–210, 300–320, and particularly 330–340 kDa and above 420 kDa. The highest *m*/*z* values of ~425 kDa clearly demonstrate that high-molecular-weight structures are formed following the modification process.

Compared to pure chitosan, the broader distribution of peaks across the *m*/*z* range and the marked increase in signal intensities in the high molecular weight region in the chitosan/GL/PEG-400-based polyol sample indicate that chain elongation and/or partial crosslinking reactions occurred in the system. Furthermore, the high intensity of peaks observed in the bio-based polyol, particularly in the ~207–212 kDa and ~299–310 kDa ranges, supports the rearrangement of macromolecular chains and the formation of more complex oligomeric/polymeric structures.

In contrast to the relatively more limited and clustered multimodal distribution of pure chitosan, the bio-based polyol sample exhibited a broader structure that was shifted toward higher molecular weights and displayed significantly increased polydispersity.

These findings suggest that simultaneous depolymerization and repolymerization processes may occur in the chitosan structure when the reaction is conducted at a high temperature of approximately 120 °C. This suggests that these processes, in conjunction with the applied chemical modification, significantly reshape the molecular architecture of chitosan and promote the formation of more complex, potentially partially cross-linked macromolecular network structures.

### 3.3. Fourier Transform Infrared Spectroscopy for Bio-Based Polyol

**Figure 4:**
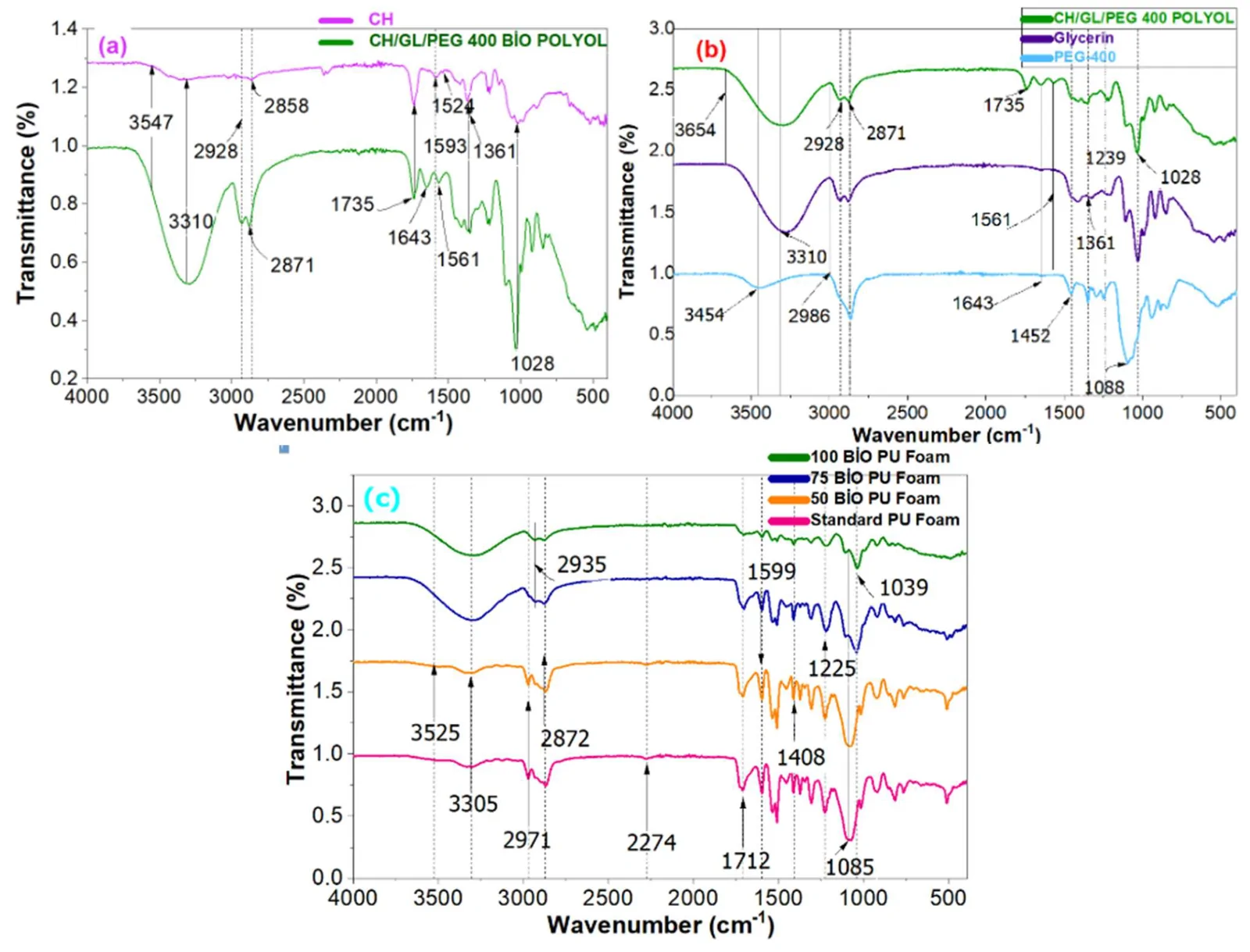
(**a**) FTIR spectra of chitosan and bio-based polyol samples based on CH/GL/PEG-400. (**b**) Comparison of FTIR spectra of CH/GL/PEG-400, GL and PEG-400. (**c**) Comparison of FTIR spectra of standard PU foam and bio-based PU foam samples.

FTIR analysis was performed to investigate the functional groups and possible changes in the molecular structure of the bio-based polyol prepared using chitosan (CH), glycerine (GL), and PEG-400. The spectra of pure chitosan, glycerine, PEG-400, and the CH/GL/PEG-400-based polyol samples are presented in a comparative manner in Figure 4a,b. The spectra were examined to identify the characteristic absorption bands of the starting components and to evaluate the changes in the positions and profiles of these bands after the polyol preparation process.

In Figure 4a, the bands observed at 3547 and 3310 cm^−1^ in the FTIR spectrum of pure chitosan can be attributed to the stretching vibrations of the hydroxyl (–OH) and amino (–NH_2_) groups, respectively. The bands at 2928 and 2858 cm^−1^ are attributed to the asymmetric and symmetric stretching vibrations of aliphatic C–H bonds, respectively. To attribute the absorption observed at approximately 1735 cm^−1^ to carbonyl (C=O) groups, the degree of acetylation of chitosan and the composition of the sample must be considered. The band at 1593 cm^−1^ is primarily associated with the bending vibrations of amino groups, while the band at 1524 cm^−1^ can be attributed to N–H bending and C–N stretching vibrations associated with amide II vibrations. The band at approximately 1361 cm^−1^ is attributed to C–H bending vibrations, while the band at 1028 cm^−1^ is attributed to the vibrations of C–O and C–O–C bonds in the polysaccharide backbone [28,31,49].

When the spectra of pure glycerine, PEG-400, and the prepared CH/GL/PEG-400-based polyol are compared in Figure 4b, differences are observed particularly in the 2986–3654 cm^−1^ region, where the stretching vibrations of the hydroxyl groups are located. The broad band observed at 3454 cm^−1^ in the PEG-400 spectrum is associated with the O–H stretching vibrations of the terminal hydroxyl groups. The broad band identified at 3310 cm^−1^ in the spectra of glycerine and the CH/GL/PEG-400-based polyol reflects the stretching vibrations of hydroxyl groups and hydrogen-bond interactions between molecules. The change in the band profile in the same region of the prepared polyol spectrum can be explained by the overlap of absorptions from different components and differences in the molecular environments of hydroxyl-containing groups. It is likely that hydrogen bond interactions occur between the hydroxyl groups of glycerine and PEG-400 and the hydroxyl and amino groups of chitosan. However, since an increase or broadening of the absorption in this region could also be expected with the addition of hydroxyl-containing components to the formulation, it cannot be interpreted on its own as evidence that chitosan has been chemically modified.

In the region where aliphatic C–H stretching vibrations are observed, a comparison of the bands at 2928 and 2858 cm^−1^ of pure chitosan with those at 2928 and 2871 cm^−1^ of the prepared polyol reveals differences in the positions of the absorption peaks, particularly in the low-wavenumber region. The presence of vibrations associated with aliphatic C–H bonds in the spectra of pure glycerin and PEG-400 suggest that the spectral profile of the prepared polyol in this region may include contributions from all three components. Therefore, the shift from approximately 2858 cm^−1^ to 2871 cm^−1^ may be related to changes in the molecular environment or may result from the overlap of characteristic bands of different components. Furthermore, when evaluating the counterpart of the band associated with the C–H vibrations of pure PEG-400 in the polyol spectrum, both the band position and its profile must be considered. However, due to the presentation of the spectra on the vertical axis and possible differences in normalization, changes in absorption intensity alone do not indicate that a chemical reaction has occurred between the components.

Spectral differences were also observed in the region associated with amino and amide groups. The fact that the band identified at 1593 cm^−1^ in pure chitosan was observed at approximately 1643 cm^−1^ in the polyol spectrum, and the band at 1524 cm^−1^ shifted to approximately 1561 cm^−1^, suggests that changes have occurred in the molecular environment where the relevant vibrations are located. It is hypothesized that these differences may be related to the bond interactions occurring between the amino and hydroxyl groups of chitosan and the hydroxyl groups of glycerine and PEG-400.

In the fingerprint region, the characteristic absorption band of pure PEG-400 observed at 1088 cm^−1^ is associated with the stretching vibrations of the C–O–C bonds in the polyether chain. The band observed at 1028 cm^−1^ in the prepared polyol may include a combined contribution from the C–O and C–O–C bonds in the chitosan polysaccharide backbone, as well as C–O vibrations originating from glycerin and PEG-400. Similarly, the band at 1239 cm^−1^ in the polyol spectrum can be attributed to the vibrations of C–O-containing groups. The fact that the band profiles in these regions differ from those of the pure components reflects the composition of the formulation and the spectral contributions of the functional groups. Furthermore, in the evaluation of the absorption band observed at 1735 cm^−1^ related to the chitosan structure, the fact that this band is not observed in the spectra of glycerine and PEG-400 but is present in the structure of the prepared bio-based polyol indicates the presence of chitosan in the polyol’s chemical structure.

Consequently, a comparison of the FTIR spectra of pure chitosan, glycerine, and PEG-400 samples with the spectrum of the CH/GL/PEG-400-based polyol prepared from these components reveals changes in the positions and profiles of the characteristic absorption bands. These changes may be related to the molecular interactions between the components in the formulation. However, because the overlap of absorption bands belonging to different components may also contribute to the observed spectral differences, the FTIR results alone do not definitively establish that chitosan’s amino or hydroxyl groups are directly involved in the chemical reaction. Therefore, to evaluate potential changes in the molecular structure of the prepared polyol in greater detail, the FTIR findings were examined in conjunction with the MALDI-TOF mass spectrometry results.

A comparison of the MALDI-TOF results presented in Table 3 and Table S1 reveals differences in molecular weight distribution between the bio-based polyol prepared with pure chitosan and the control. The observed changes suggest that differences in molecular structure or distribution of molecular species within the sample occurred following the polyol preparation process. When evaluated in conjunction with the FTIR findings, the MALDI-TOF results support the possibility that interactions may have occurred between chitosan, glycerine, and PEG-400. However, since the differences in molecular weight distribution do not, by themselves, prove the formation of covalent bonds, these results should be interpreted as supportive evidence of possible chemical modification.

### 3.4. Fourier Transform Infrared Spectroscopy for PU Foams

FTIR analysis was performed to determine the changes in the chemical structure of polyurethane foams produced using CH/GL/PEG-400-based biopolyol instead of fossil-derived polyether polyol, and to highlight the structural differences compared to standard PU foam. The FTIR spectra of the samples are shown in Figure 4c.

Upon examination of Figure 4c, the broad bands observed at wavenumbers of 3535 and 3305 cm^−1^ in all foam samples correspond to the stretching vibrations of free and hydrogen-bonded hydroxyl (O–H) groups and the N–H groups in the urethane structure, respectively [19]. While the intensities of these bands were similar in the standard PU and 50 BIO PU foam samples, a significant increase in both the intensity and width of the relevant bands was observed as the bio polyol content in the system increased (75 BIO PU and 100 BIO PU). This is due to the increase in hydroxyl groups introduced into the system by the addition of bio polyol and the resulting strengthening of the hydrogen-bond interactions in the urethane matrix.

The bands appearing in the 2971 and 2872 cm^−1^ regions of the spectra represent the asymmetric and symmetric stretching vibrations of the aliphatic –CH_2_ and –CH_3_ groups, respectively. In parallel with the increase in the bio polyol ratio, the band at 2971 cm^−1^ shifted to approximately 2935 cm^−1^, particularly in the 75 BIO PU and 100 BIO PU foam samples [12]. This shift toward a lower wavenumber is believed to result from changes in the chemical environment of the polymer chains, as well as from the structural differences in the bio polyol and altered hydrogen-bonding dynamics.

The characteristic band observed at approximately 1712 cm^−1^ in all foam samples corresponds to the stretching vibrations of the carbonyl (C=O) groups in the urethane structure. Additionally, bands corresponding to the C–O stretching vibrations in the urethane bond were clearly observed in the 1225 cm^−1^ region. The C–O vibrations appearing at approximately 1085 cm^−1^ in the Standard PU and 50 BIO PU samples shifted to 1039 cm^−1^ in the 75 BIO PU and 100 BIO PU samples [22]. This shift supports the modifications occurring in the chemical environment of the polyurethane network structure owing to the incorporation of bio polyols, as well as intermolecular differences in hydrogen-bond interactions. Furthermore, the evaluation of the C–O/C–O–C vibrations in the 1039–1085 cm^−1^ region, together with the C–N vibrations observed around 1408 cm^−1^, supports the formation of urethane bonds through the reaction of hydroxyl groups with isocyanate groups [32]. These findings demonstrate that the synthesized CH/GL/PEG-400-based bio-based polyol was successfully incorporated into the polyurethane network structure.

On the other hand, the characteristic band at 2274 cm^−1^ associated with free isocyanate (–NCO) groups [30] was observed at low intensity in the standard PU foam and 50 BIO PU Foam samples, whereas this band was not detected in the 75-BIO PU Foam and 100 BIO PU Foam samples. The disappearance of the free –NCO band in the samples containing a high proportion of bio-based polyol suggests that the isocyanate groups were largely consumed by hydroxyl groups during the reaction and that the urethane formation reaction occurred with high conversion. However, considering that FTIR analysis cannot quantitatively determine the reaction conversion, this result should be evaluated solely as a qualitative finding that supports the formation of urethane bonds.

In conclusion, the FTIR findings demonstrate that the CH/GL/PEG-400-based bio polyol was successfully chemically integrated into the polyurethane network structure, and an increase in the bio polyol ratio led to significant modifications in the hydrogen bonds and chemical environment of the polymer matrix. These data strongly support the notion that the synthesized bio polyol can be successfully used in polyurethane foam production as a sustainable alternative to conventional fossil-based polyether polyols.

### 3.5. Scanning Electron Microscopy

**Figure 5:**
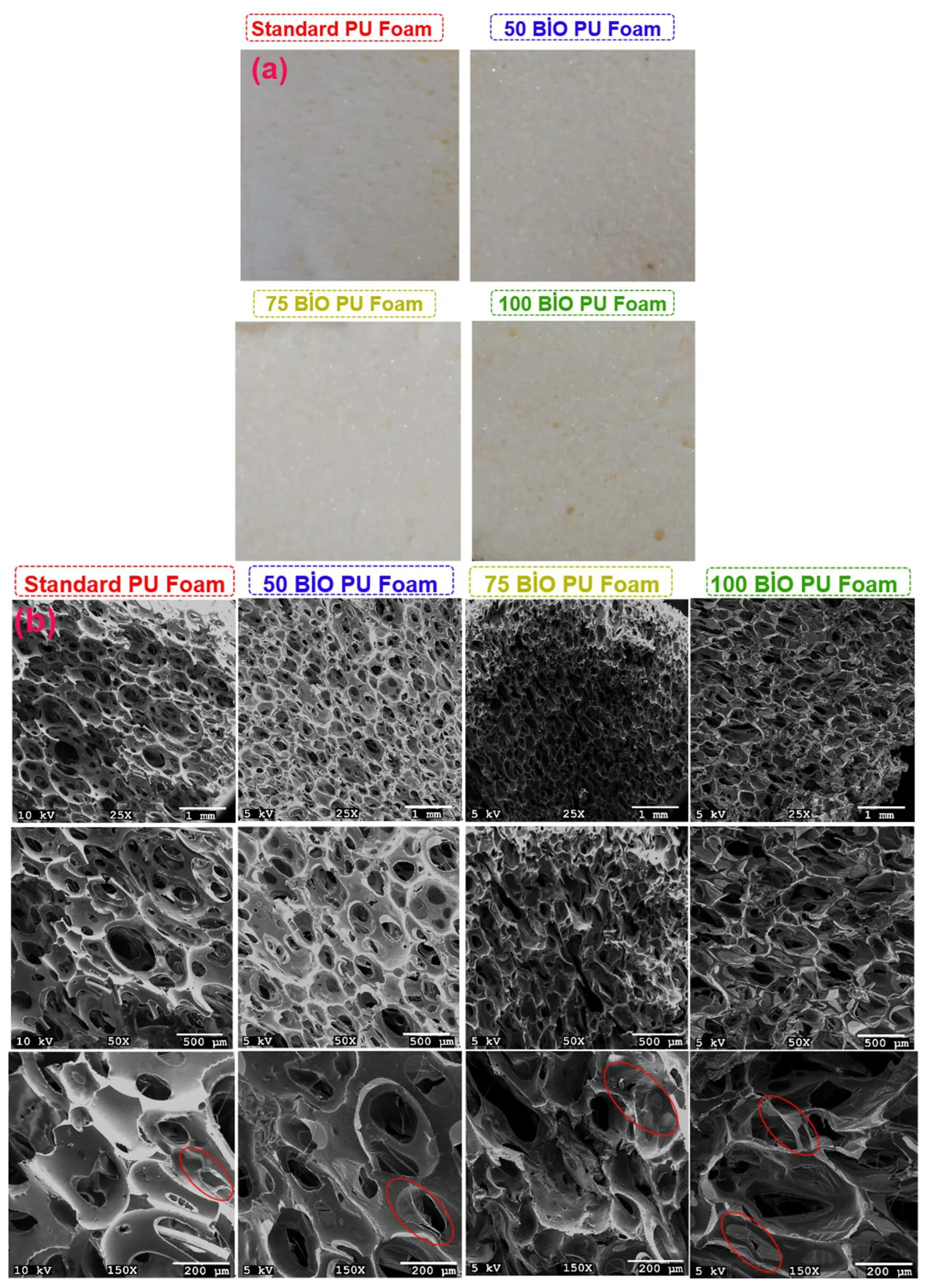
(**a**) Macroscopic images of standard PU foam and bio-based polyol-based PU foam samples, and (**b**) SEM micrographs obtained from the regions marked in the macroscopic images.

SEM analysis was performed to examine the cellular morphology and pore structures of polyurethane foams produced using standard PU foam and bio-based polyols based on CH/GL/PEG-400 at different ratios. The results are shown in Figure 5. Figure 5a shows the foam samples prepared for SEM analysis, and Figure 5b presents the SEM images of these samples.

Comparing the SEM images revealed that certain morphological changes occurred in the foam cell structure as the bio-based polyol content increased. In particular, it was observed that in samples with high bio-based polyol content, the cell sizes decreased, and the cells exhibited a more homogeneous distribution. In the 100-BIO PU Foam sample produced using 100% bio-based polyol, a cellular structure with smaller cell diameters and a more uniform distribution was formed compared to the other samples. Conversely, as the amount of bio-based polyol increased, thinner cell walls and more delicate cell membranes were observed in the open cell regions, as indicated by the red circles in Figure 5b.

These morphological changes are thought to result from the macromolecular structure of the bio-based polyol, which increases the viscosity of the reaction medium. The macromolecular structure of the bio-based polyol and the resulting increase in mixture viscosity may support the formation of more ordered, finer, and more homogeneous cellular structures by limiting the growth and coalescence of gas bubbles during foaming. Consequently, it was assessed that the cell walls developed more stably, and the cellular structure exhibited a more controlled morphology. The SEM findings obtained are consistent with the recent literature results reporting that high-viscosity bio-based polyols improve the cellular structure of polyurethane foams and promote the formation of a more homogeneous cell morphology [50].

To examine the surface morphology of the Standard PU, 50BioPU, 75BioPU, and 100BioPU foam samples, quantitative image analysis was performed on cross-sectional areas of approximately 3.5 mm × 3.5 mm, using a 1 mm scale bar as a reference; the open-cell fractions were 86.7%, 87.1%, 84.7%, and 86.2%, respectively. These findings confirm that the cellular structure of the developed bio-based polyurethane foams is highly similar to the morphological characteristics of standard fossil-based polyurethane foam.

Overall, no significant deterioration or irregularities were observed in the cellular morphologies of the standard PU foam, 50BioPU, 75BioPU, and 100BioPU foam samples. Furthermore, quantitative image analysis confirmed that the open-cell content remained remarkably stable across the series, ranging from 84.7% to 87.1%. The fact that a regular and homogeneous cellular structure, along with a well-maintained open-cell ratio, was largely preserved despite the incorporation of the bio-based polyol strongly supports the notion that the synthesized CH/GL/PEG-400-based bio-based polyol can successfully serve as a sustainable alternative polyol in the production of semi-rigid and flexible polyurethane foams.

### 3.6. Water Absorption

**Figure 6:**
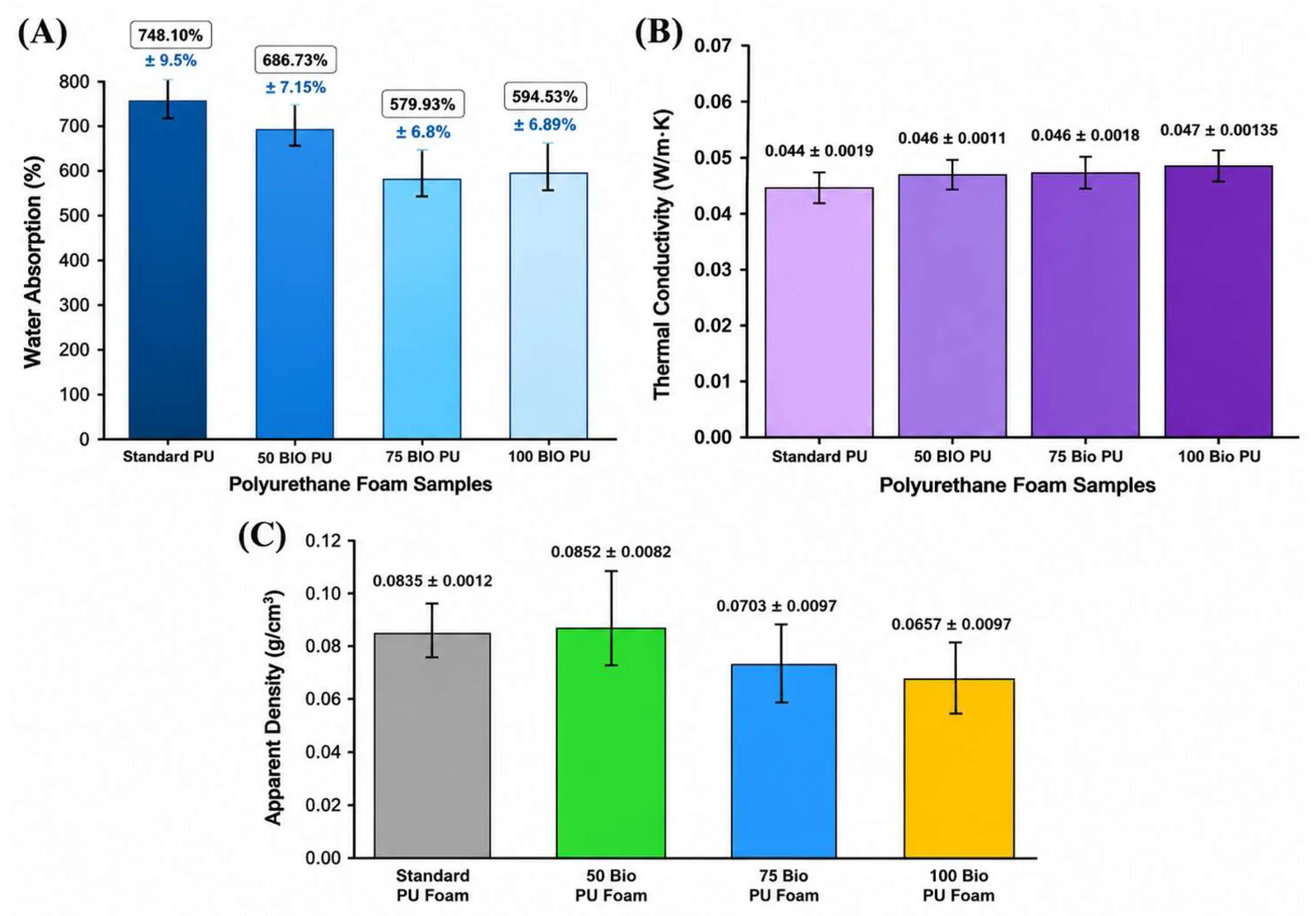
(**A**) Water absorption capacity, (**B**) thermal conductivity and (**C**) apparent density results for standard PU foam and samples of 50 BIO PU Foam, 75 BIO PU Foam, and 100 BIO PU Foam.

To evaluate the performance of polyurethane foams produced using the synthesized CH/GL/PEG-400-based bio-derived polyol in humid environments, the water absorption capacities of standard PU foam and samples of 50 BIO PU Foam, 75 BIO PU Foam, and 100 BIO PU Foam were determined under the same experimental conditions. The results are shown in Figure 6A.

The mass water absorption capacity of standard PU foam was determined to be 748.10%, and the values of the samples were 686.73%, 579.93%, and 594.53% for the 50-BIO PU, 75-BIO PU, and 100-BIO PU foam samples, respectively. Accordingly, compared with standard PU foam, the mass water absorption capacities decreased by 8.20%, 22.48%, and 20.53%. Furthermore, the apparent density slightly increased from 0.0835 g/cm^3^ for standard PU to 0.0852 g/cm^3^ for 50-BIO PU and then decreased to 0.0703 g/cm^3^ for 75-BIO PU and 0.0657 g/cm^3^ for 100-BIO PU. Expressing water absorption in polymeric foams solely as a percentage of the initial dry mass (mass percentage, %) may not be sufficient for a complete comparison among samples with different apparent densities, because lower-density matrices tend to show artificially higher percentages on a mass basis. Considering this, the volumetric water absorption evaluation confirmed that bio-based polyols also limit water retention capacity on a per-unit-volume basis. This improvement in water absorption capacity was attributed to both changes in the chemical network structure and the foam cellular morphology. SEM and quantitative image analyses showed that all samples retained a high degree of open-cell structure (ranging from 84.7% to 87.1%). In contrast, as the bio-based polyol content increased, the average cell size decreased, and the cell structure became more homogeneous. The decrease in water absorption capacity, despite the lower apparent density (higher void fraction), can be explained by the high capillary resistance caused by the shrinking cell sizes and the resulting hindrance to the diffusion of water molecules within the matrix. Furthermore, FTIR analyses indicated that in samples with a high bio-based polyol content, the O–H and N–H bands became more pronounced, suggesting that the hydrogen bonding interactions were strengthened [51]. These intermolecular interactions render the polyurethane matrix more compact, thereby reducing the specific free volume of the polymer matrix and making it more difficult for water molecules to penetrate the cell walls. The similarity in the water absorption values of the 75-BIO PU and 100-BIO PU samples indicates that the polymer network structure and morphological compaction have reached a certain threshold value.

In conclusion, when apparent density, cellular morphology, and mass/volume water retention parameters are evaluated together, it has been demonstrated that the use of CH/GL/PEG-400-based bio-based polyols clearly increases the water resistance of polyurethane foams.

### 3.7. Thermal Conductivity

Thermal conductivity analyses were conducted to evaluate the thermal insulation performance of polyurethane foams produced using the synthesized CH/GL/PEG-400-based bio-polyol and to compare them directly with standard fossil-based PU foams. Thermal conductivity is a crucial parameter that dictates the insulation efficiency and determines the suitable application areas. In this study, thermal conductivity measurements were evaluated at an elevated temperature (100 °C) to justify the thermal insulation integrity and structural stability of the developed foams under demanding operational conditions, such as building envelopes subjected to intense solar radiation (roofs and facades), hot pipeline insulation, and industrial heat shielding. The results are shown in Figure 6B.

According to the results, the thermal conductivity of the standard PU foam sample was 0.044 W m^−1^ K^−1^. For the 50-BIO PU Foam, 75-BIO PU Foam, and 100-BIO PU Foam samples, these values were measured as 0.046, 0.046, and 0.047 W m^−1^ K^−1^ respectively. Accordingly, compared to the standard PU foam, there was a slight increase in the thermal conductivity of 4.55%, 4.55%, and 6.82%, respectively.

Nevertheless, the fact that the thermal conductivity values of all bio-based formulations remained within a narrow range of 0.046 ± 0.0011 to 0.047 ± 0.00135 W m ^−1^ K ^−1^ at an elevated testing temperature (100 °C), showing no statistically significant deviation from the Standard PU control (0.044 ± 0.0019 W m ^−1^ K ^−1^), demonstrates that the incorporation of the CH/GL/PEG-400 bio-polyol preserves the high-temperature thermal insulation performance. The observed minor increase is attributed to the partial impact on the heat transfer mechanism in the gas phase, resulting from subtle changes in the foam’s cellular morphology and variations in the polymer network structure caused by the addition of the bio-based polyol [52].

In conclusion, the use of the synthesized CH/GL/PEG-400-based bio-based polyol increased the bio-based content of the polyurethane foams while causing only limited changes in thermal conductivity values, and it was demonstrated that all the foams obtained exhibited acceptable thermal performance for thermal insulation applications. These results indicate that the synthesized bio-based polyol can serve as a sustainable alternative to traditional fossil-based polyols.

### 3.8. Apparent Density Test

Apparent density analyses were conducted on five replicates of Standard PU Foam, 50 Bio PU Foam, 75 Bio PU Foam, and 100 Bio PU Foam samples, and the resulting average values are presented in Figure 6C. According to the results, the apparent density of the Standard PU Foam sample was determined to be 0.0835 g/cm^3^, whereas the values for the 50 Bio PU Foam, 75 Bio PU Foam, and 100 Bio PU Foam samples were calculated as 0.0852, 0.0703, and 0.0657 g/cm^3^, respectively. Overall, a clear trend of decreasing apparent density in the polyurethane foams was observed as the bio-based polyol content increased.

This change in the apparent density can be attributed to the effect of the bio-based polyol on the foam formation mechanism. The hydroxyl groups present in the bio-based polyols react more effectively with isocyanate groups, contributing to the formation of the polyurethane network structure. Gas formation, which occurs simultaneously with the formation of the cross-linked structure as the reaction progresses, and the trapping of this gas within the cell structure are among the key parameters affecting foam expansion. Therefore, the increase in bio-based polyol content, along with the resulting increase in foam volume and the corresponding decrease in the amount of solid phase per unit volume, may have caused the apparent density to decrease [7,53].

This assessment was further supported by microstructural observations obtained from SEM analyses. SEM images revealed that foams containing bio-based polyols exhibited a more regular and homogeneous cellular morphology than standard polyurethane foam. Furthermore, when the finer and more homogeneous cell structure observed in the SEM images is evaluated in conjunction with the decrease in apparent density, it can be concluded that the use of bio-based polyol positively affects the foam formation process and the development of the cellular structure of the foam.

Also, the findings from the FTIR analyses shown in Figure 4b also support this interpretation. In foam samples with an increased bio-based polyol content, the characteristic absorption band at 2274 cm^−1^ associated with free isocyanate groups was significantly reduced or completely absent. This indicates that the hydroxyl groups present in the bio-based polyols reacted extensively with the isocyanate groups, leading to a more effective formation of the polyurethane network structure. The reaction proceeding with a higher degree of conversion may explain the production of foams with higher expansion ratios and, consequently, lower apparent densities, as it contributes to more effective gas trapping within the cell structure during foam formation.

In conclusion, the use of bio-based polyols contributes to the use of sustainable raw materials and enables the production of polyurethane foams with lower apparent densities by influencing foam morphology. When the measured apparent density is evaluated in conjunction with the SEM and FTIR results, it becomes clear that bio-based polyols play a significant role in the development of the cellular structure of polyurethane foams and their expansion behavior.

### 3.9. Thermogravimetric Analysis

**Figure 7:**
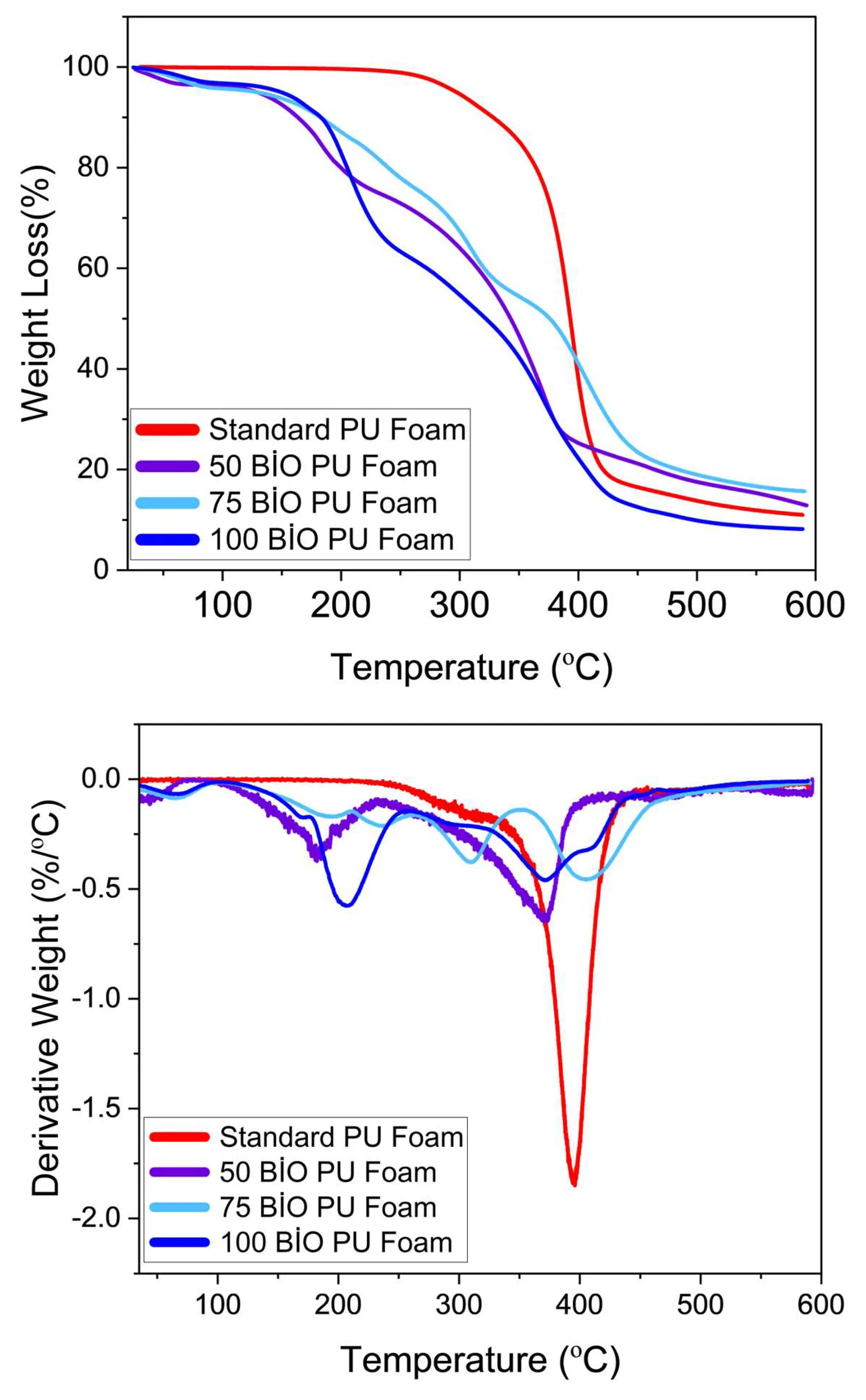
TGA and DTG curves for the Standard PU Foam, 50 Bio PU Foam, 75 Bio PU Foam, and 100 Bio PU Foam samples, which contain a bio-based polyol based on CH/GL/PEG-400, compared to standard PU foam.

The thermal decomposition behaviors of the 50 Bio PU Foam, 75 Bio PU Foam, and 100 Bio PU Foam samples produced using a bio-based polyol based on CH/GL/PEG-400 in place of standard PU foam were investigated via TGA and DTG analyses, and the results are presented in Figure 7 and Table 4.

As summarized in Table 4, the standard PU foam exhibited the typical two-stage thermal decomposition behavior commonly reported in the literature for conventional polyurethanes. For the standard PU foam, the first decomposition stage began at the 5% mass loss temperature (T__%5_) of 293 °C and T__%10_ at 325 °C; it passed through the temperatures T__%15_ (350 °C) and T__%40_ (387 °C), peaking at the maximum decomposition rate temperature (T__%max_) of 397 °C. While this primary decomposition stage is primarily attributed to the thermal breakdown of urethane and urea bonds (hard segments), the second stage corresponds to the decomposition of polyether-based soft segments [12,21]. At the end of the analysis, the standard PU foam left behind 11.00% ash residue.

In contrast, the addition of the synthesized CH/GL/PEG-400-based bio-polyol significantly altered the thermal decomposition profile and revealed three distinct decomposition regions. The initial minor mass loss observed in the bio-based foams at approximately 100 °C was related to the removal of physically adsorbed moisture owing to the hydrophilic nature of the bio-based polyol. More importantly, the initial thermal stability, represented by T__%5_, T__%10_, and T__%15_, shifted to significantly lower temperatures with the addition of the bio-polyol. For example, for the 50 Bio PU, 75 Bio PU, and 100 Bio PU foams, the T__%5_ values decreased to 129 °C, 129 °C, and 149 °C, respectively, while the T__%10_ values were recorded as 163 °C, 183 °C, and 183 °C, respectively (Table 4). This mass loss in the early stage is a phenomenon commonly observed in bio-based systems and stems from the thermal degradation of low-molecular-weight components that cannot fully incorporate into the network structure or of unreacted oligomeric fractions [32,33].

Despite this decrease in early-stage decomposition temperatures (T__%5_—T__%15_), the main polymer network decomposition assessed based on T__%40_ and the maximum decomposition temperature (T__%max_) exhibited interesting behavior depending on the formulation. The T__%40_ values were observed to be 315 °C for 50 Bio PU, °C for 75 Bio PU, and 272 °C for 100 Bio PU. In particular, the maximum degradation temperature (T__%max_) of the 75 Bio PU foam reached 408 °C, which is even higher than that of standard PU foam (397 °C). This indicates that the optimal 75% bio-polyol substitution creates a synergistic cross-linking density that enhances the main chain’s thermal resistance. However, the complete replacement of the petroleum-based polyol (100 Bio PU) resulted in a lower T__%max_ value of 388 °C, indicating structural limitations that arise when the traditional soft segment is absent [33].

Char yields at 600 °C varied significantly among the formulations, highlighting the effect of bio-polyol on carbonization. While the standard PU foam yielded 11.00% carbon residue, the 50 Bio PU and 75 Bio PU foams exhibited increased carbon yields of 12.86% and 15.68%, respectively. The superior char formation in the 75 Bio PU formulation stems from a carbonized protective layer promoted by the ring structures of chitosan and the polyol main chain that acts as a barrier against heat and mass transfer during combustion [32,33]. In contrast, the 100 Bio PU foam exhibited a lower char residue of 8.17%; this suggests that the excessive concentration of the bio-based polyol negatively affected the formation of a stable and balanced cross-linked carbon matrix under high-temperature conditions.

In conclusion, quantitative TGA/DTG data clearly demonstrate that the inclusion of CH/GL/PEG-400-based bio polyol lowers the initial decomposition temperatures owing to the presence of weaker/more volatile bio-based bonds; however, an optimized blending ratio (e.g., 75% Bio PU) successfully increases the maximum thermal decomposition temperature (T__max%_) and the formation of char residue, thereby altering the degradation pathway and improving high-temperature carbonization performance.

### 3.10. UL-94 Vertical Burning Test

To evaluate the flame behaviour of polyurethane foams produced from CH/GL/PEG-400-based bio-based polyols, the UL-94 vertical burning test was conducted on Standard PU Foam, 50 Bio PU Foam, 75 Bio PU Foam, and 100 Bio PU Foam samples. The test results showed that all the foam samples continued to burn after 10 s of flame contact and did not exhibit self-extinguishing behaviour. Additionally, molten polymer droplets were formed during combustion, which ignited the cotton indicator.

Based on these findings, all the foam samples examined were classified as V-2 flammability class according to the UL-94 standard. The results indicate that the use of bio-based polyols based on CH/GL/PEG-400 alone is insufficient to elevate the flame-retardant performance of polyurethane foams to the V-0 or V-1 level. The primary reason for this is that although the bio-based polyol alters the chemical structure of the polyurethane matrix, it does not contain active flame-retardant components capable of forming a protective char layer during combustion or providing a radical-quenching mechanism in the gas phase.

### 3.11. Compression Test

**Figure 8:**
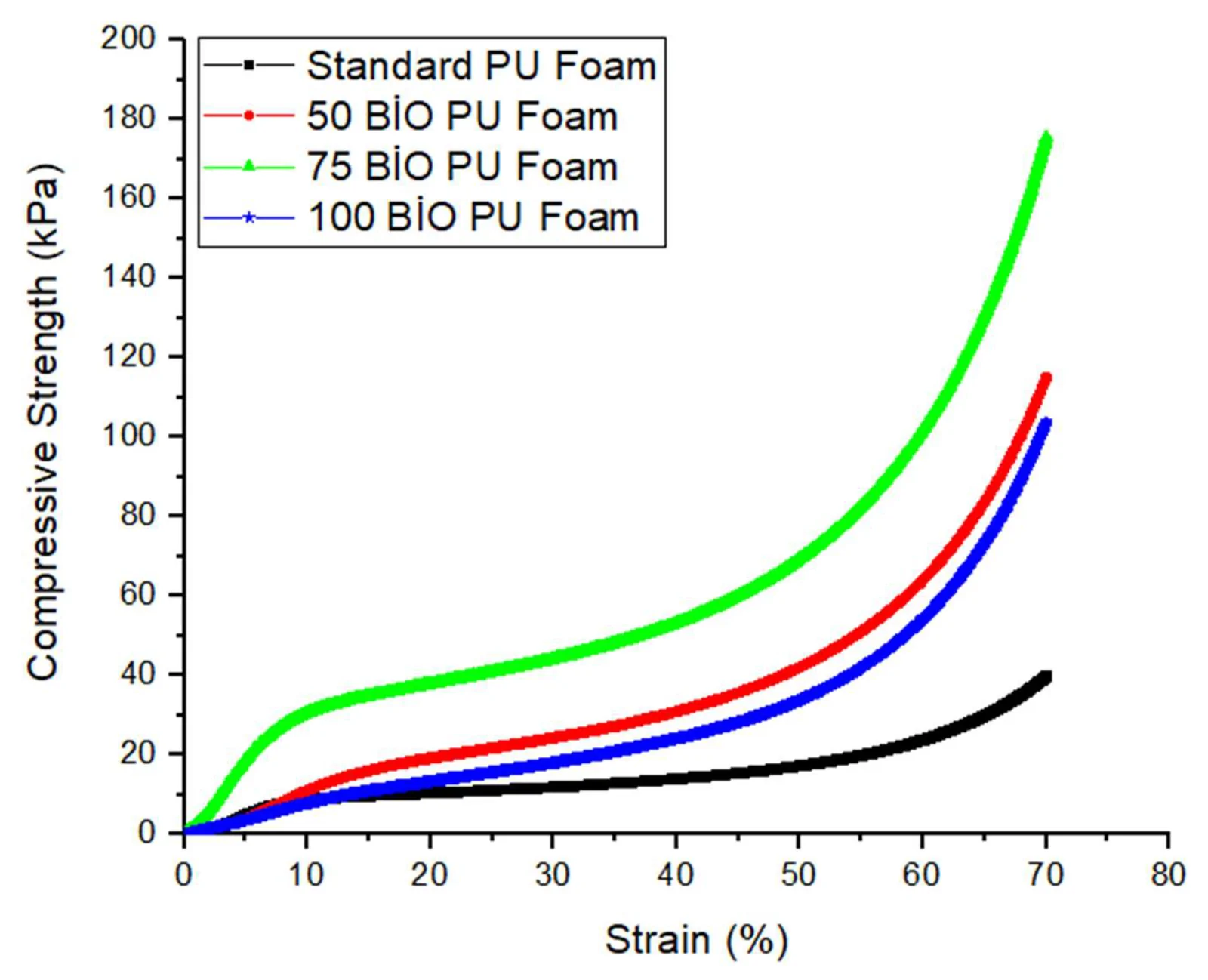
Compressive strength–strain curves of the Standard PU Foam, 50 Bio PU Foam, 75 Bio PU Foam, and 100 Bio PU Foam samples.

Figure 8 illustrates the compressive stress–strain behaviour of the Standard PU, 50 Bio PU, 75 Bio PU, and 100 Bio PU Foam samples up to 70% strain, revealing distinct differences in load-bearing capacity that are directly related to the crosslink density and cellular structure imparted by the CH/GL/PEG-400-based bio polyol at each substitution ratio. The stress–strain curve of the Standard PU Foam shows the lowest overall response among the four formulations. A short, low-modulus linear-elastic region (cell-wall bending) is followed by an early and relatively flat plateau stage, after which densification begins only at high strain. This behaviour is consistent with a foam network built exclusively on the petroleum-based polyether polyol (OH number = 57 mg KOH/g), which produces a comparatively lower crosslink density and a less rigid cell-wall structure. Substitution of 50% of the conventional polyol with the CH/GL/PEG-400-based bio polyol nearly triples the compressive strength relative to the Standard sample. The curve displays an initial elastic slope comparable to that of the Standard sample (≈120 kPa); however, the elastic–plateau transition is shifted to a higher strain (≈14%) and stress (≈15 kPa). This enhancement is attributable to the higher hydroxyl functionality of the bio polyol (OH number = 87 mg KOH/g), which increases the crosslink density of the urethane network and stiffens the cell walls. The 75 Bio PU Foam sample exhibits the highest compressive strength of the entire series (≈4.4-fold that of the Standard sample). The curve shows the steepest elastic region (E ≈ 430 kPa) and the highest plateau stress; densification begins at a strain similar to that of the other formulations but at a markedly higher stress level, indicating that 75% substitution represents the optimum balance between crosslink density, cell-wall thickness, and cell homogeneity in this system. Although it is still more durable than standard PU foam, in the case of a full replacement (100%), a noticeable decrease in compressive strength is observed compared to both the 50% and 75% samples. Compared with the 75 Bio PU sample, cell-wall collapse occurs at a similar or even slightly higher strain (≈10% vs. ≈8%) but at a much lower stress (≈8 vs. ≈26 kPa), and the curve shows a lower initial modulus (≈90 vs. ≈430 kPa) and a lower plateau stress. Although SEM analyses revealed that the 100 Bio PU foam has the smallest cell diameter and a highly homogeneous cell size distribution, this decline in mechanical performance is attributed to changes in the matrix’s chemical and physic mechanical structure rather than to cellular morphological degradation [54].

Increasing the bio polyol ratio to 100% excessively increased the crosslinking density in the matrix, thereby restricting the flexibility of the polymeric network and imparting significant brittleness to the structure. Furthermore, the high-molecular-weight and branched macromolecular architecture of the bio polyol can lead to steric hindrance at high substitution ratios, negatively affecting optimal hard/soft segment phase separation and isocyanate accessibility in the final product. This situation, despite the homogeneous cellular morphology, facilitates microcrack propagation within the matrix under compressive stresses and leads to cell-wall collapse at a low stress level (≈8 kPa) and a reduced plateau stress [55,56,57].

The non-monotonic course of compressive strength observed in this study, an increase from standard PU foam to 75% bio-PU foam, followed by a decrease at a 100% substitution level, is consistent with the mechanisms reported for other bio polyol-modified polyurethane foam systems. Analysis of the mechanical properties of bio polyol-modified polyurethane-polyisocyanurate systems has shown that the normalized compressive strength increases significantly as the bio polyol content increases, owing to the incorporation of a greater cross-linked structure; this effect is attributed to the higher hydroxyl value and functionality that bio-based polyols can impart to the network compared to traditional petrochemical polyols [54]. Similarly, in rigid polyurethane foams modified with rapeseed oil-derived bio polyols of varying functionality, the addition of bio polyols improves the cellular structure by reducing cell size. This finding confirms that the improvement in cell homogeneity directly contributes to the mechanical strengthening observed at moderate substitution levels, such as 50% and 75%, in the current formulations [58,59].

However, mechanical degradation at a 100% substitution level parallels the phenomena of brittleness and restricted chain mobility, often attributed to excessive cross-linking, commonly reported in rigid polyurethane systems containing high levels of bio polyol [53]. Consequently, the complete replacement of conventional polyol with 100% Bio PU Foam, despite preserving cellular homogeneity, exhibited lower compressive strength than the 75% formulation because the excessive bio polyol content disrupted the ideal hard/soft segment balance and increased the matrix brittleness [50,51,52,53,54].

To provide a more detailed quantitative comparison among the formulations, characteristic compressive parameters were extracted from the averaged stress–strain curves in Figure 8 (*n* = 5) and are summarised in Table 5, together with the NCO/OH index, apparent density (ρ) and open-cell content of each foam. These parameters include the apparent elastic modulus (E, slope of the initial linear region of the stress–strain curve); the strain and stress at the elastic–plateau transition (collapse point, ε_c_ and σ_c_, determined from the intersection of the tangents to the linear-elastic and plateau regions); and the compressive stresses at 10% and 50% strain (σ_10_ and σ_50_).

Values were derived from the averaged stress–strain curves (*n* = 5) in Figure 8. E: slope of the initial linear region; ε_c_/σ_c_: two-tangent intersection of the linear-elastic and plateau regions. Compressive strength was taken as the stress at 10% relative deformation (ISO 844), since no stress maximum occurred below this deformation. Apparent density values are given as mean ± standard deviation (Figure 6C).

As seen in Table 5, the formulations differ markedly in the small- and intermediate-strain regimes. The 75 Bio PU foam was the only formulation exhibiting a well-defined linear-elastic region and a distinct collapse point; cell-wall collapse occurred at ≈8% strain under ≈26 kPa, i.e., at more than three times the collapse stress of the Standard foam (≈8 kPa at ≈7% strain). Consequently, the 75 Bio PU foam already carried ≈30 kPa at 10% strain, whereas the other three formulations remained within 8–11 kPa at the same strain. The elastic–plateau transition of the 50 Bio PU foam was shifted to a higher strain (≈14%) and stress (≈15 kPa). In contrast, the stress of the 100 Bio PU foam remained slightly below that of the Standard foam up to ≈12% strain, indicating early elastic buckling/fracture of the thin cell walls; beyond this strain, its curve rose above that of the Standard foam. At 50% strain, the stresses were ≈17, ≈42, ≈69 and ≈34 kPa for the Standard, 50, 75 and 100 Bio PU foams, respectively, corresponding to 2.5-, 4.1- and 2.0-fold increases relative to the Standard foam for the bio-based formulations.

In terms of the standard mechanical parameters, the compression modulus (E) increased from ≈120 kPa for the Standard foam to ≈430 kPa for the 75 Bio PU foam (≈3.6-fold), remained unchanged for the 50 Bio PU foam (≈120 kPa) and decreased to ≈90 kPa for the 100 Bio PU foam. Similarly, the compressive strength at 10% deformation (σ_10_, ISO 844) was ≈8, ≈11, ≈30 and ≈8 kPa for the Standard, 50, 75 and 100 Bio PU foams, respectively, corresponding to 1.4- and 3.8-fold increases for the 50 and 75 Bio PU foams and no change for the 100 Bio PU foam compared with the Standard foam. At the end of the test (70% strain), the compressive stress reached ≈40, ≈115, ≈175 and ≈104 kPa, respectively; the highest value (≈175 kPa, 75 Bio PU) corresponds to the compressive strength reported in the Abstract and Conclusion. The ranking of the formulations was therefore consistent for all parameters (75 Bio PU > 50 Bio PU ≥ 100 Bio PU ≥ Standard PU), except in the small-strain region, where the 100 Bio PU foam showed a slightly lower modulus than the Standard foam owing to its lower density and thinner cell walls.

The apparent elastic modulus, which governs the load-bearing capacity and dimensional stability of the foams at small deformations (e.g., under service loads in insulation applications), provides further insight into the role of the bio-polyol. In the linear-elastic region, the deformation of predominantly open-cell foams is controlled by the bending of the cell struts, with an additional contribution from the stretching of cell-face membranes when closed or partially closed cells are present. The Standard and 50 Bio PU foams exhibited practically identical moduli (≈120 kPa), indicating that replacing half of the polyether polyol did not alter the initial stiffness of the cellular structure, although it markedly increased the stress level beyond the collapse point. The 75 Bio PU foam showed a modulus of ≈430 kPa, i.e., ≈3.6 times that of the Standard foam, whereas the 100 Bio PU foam exhibited the lowest modulus of the series (≈90 kPa), ≈25% lower than that of the Standard foam and ≈79% lower than that of the 75 Bio PU foam. Thus, E does not change monotonically with the bio-polyol content but reaches a sharp maximum at 75% substitution. Notably, the ≈3.6-fold increase in stiffness of the 75 Bio PU foam was not accompanied by a reduction in the collapse strain (≈8% vs. ≈7% for the Standard foam), indicating that the bio-polyol stiffened the cell walls without making them prematurely unstable at small strains.

The modulus cannot be explained by the apparent density alone. According to the Gibson–Ashby model for open-cell foams, the relative modulus scales approximately with the square of the relative density (E*/E_s_ ∝ (ρ*/ρ_s_)^2^). On this basis, the 75 and 100 Bio PU foams, whose densities are 15.8% and 21.3% lower than that of the Standard foam, would be expected to have moduli of only ≈71% and ≈62% of the Standard value, whereas the 50 Bio PU foam (+2% density) would be expected to show a ≈4% higher modulus. The measured values instead correspond to ≈100%, ≈358% and ≈75% of the Standard modulus, respectively. When the density effect is removed (E/ρ^2^, normalised to the Standard foam), the intrinsic stiffening factors become ≈0.96, ≈5.1 and ≈1.2 for the 50, 75 and 100 Bio PU foams, respectively. Similarly, the specific modulus (E/ρ) was ≈1.44, ≈1.41, ≈6.12 and ≈1.37 kN·m/kg for the Standard, 50, 75 and 100 Bio PU foams, respectively. Relative to the Standard foam, the lower modulus of the 100 Bio PU foam can therefore be attributed largely to its lower density, since its density-normalised stiffness is even slightly higher. Relative to the 75 Bio PU foam, however, the ≈6.5% lower density of the 100 Bio PU foam would account for only a ≈13% reduction in modulus, whereas the measured reduction was ≈79%, showing that the loss of stiffness at full substitution stems mainly from changes in the polymer matrix and cell-wall structure. Conversely, the exceptionally high density-normalised modulus of the 75 Bio PU foam reflects a genuine increase in the stiffness of the cell-wall material and/or a change in the deformation mechanism of the cells.

The cellular morphology is consistent with these trends. SEM analysis showed that the bio-polyol progressively refined the cell size and improved cell homogeneity, while the open-cell content remained within 84.7–87.1%. The 75 Bio PU foam combined refined, homogeneous cells with the lowest open-cell content (84.7%, i.e., the highest fraction of closed/partially closed cells, ≈15.3% vs. 12.9–13.8% for the other foams). Because the contribution of membrane stretching scales linearly, rather than quadratically, with relative density, even a moderate increase in the fraction of intact cell faces raises the modulus disproportionately at low densities, which explains the distinct linear-elastic region and the highest E of this formulation. In the 100 Bio PU foam, although the cells were the smallest and most uniform, SEM images revealed thinner cell walls and more delicate membranes. Together with the lowest density (0.0657 g/cm^3^), this reduces the thickness-to-length ratio (t/l) of the struts, which controls their bending stiffness (E* ∝ (t/l)^4^ for open cells), and results in the lowest modulus and σ_10_ of the series. The advantage of the fine morphology of this foam therefore becomes effective only at larger strains, where the progressive compaction of the fine cells raises the stress above that of the Standard foam.

The NCO/OH index (Table 2) provides an additional chemical explanation for the non-monotonic variation in E. The Standard foam, despite a high index (1.75), is built on a low-functionality polyether polyol (OH number = 57 mg KOH/g) that forms long, flexible soft segments and a low cross-link density, resulting in a low modulus; thus, a large NCO excess alone does not guarantee a stiff network. The 50 Bio PU foam had the lowest index (1.58) because the bio-polyol introduced additional hydroxyl groups at a constant isocyanate content (Table 1). The cross-links provided by the multifunctional, branched bio-polyol (OH number = 87 mg KOH/g) appear to compensate for the reduced NCO excess, so that the modulus remained at the Standard level (≈120 kPa), while the collapse point shifted to a higher strain (≈14%) and stress (≈15 kPa), i.e., a stronger but still compliant network. Increasing the isocyanate content in the 75 Bio PU formulation raised the index to 1.80; the combination of excess NCO (additional urea, allophanate and biuret linkages that increase the hard-segment content), the multifunctional bio-polyol and the 25% residual polyether polyol, which still supplies flexible soft segments, provided the most favourable hard/soft segment balance and yielded a ≈3.6-fold higher modulus despite a 16% lower density than the Standard foam. A further marginal increase in the index to 1.83 in the 100 Bio PU foam, combined with the complete removal of the polyether soft segment, shifted the network towards an over-cross-linked, brittle state in which the thin cell walls fracture rather than bend elastically, leading to the lowest modulus despite the highest index. Therefore, the compressive behaviour, and particularly the elastic modulus, is not governed by the NCO/OH index alone but by the interplay between the index, polyol functionality/soft-segment content, apparent density and cell-wall morphology, with the optimum balance achieved at 75% bio-polyol substitution (NCO/OH ≈ 1.80, ρ = 0.0703 g/cm^3^).

## 4. Conclusions

In this study, an eco-friendly bio-based polyol was successfully synthesized using chitosan (CH), glycerine (GL), and PEG-400, and utilized to produce semi-rigid bio-based PU foams by replacing petroleum-based polyol at various substitution ratios. The chemical structure, cellular morphology, apparent density, gravimetric and volumetric water absorption behaviour, mechanical performance, thermal conductivity, thermal degradation mechanism via TGA/DTG, and vertical burning behaviour of the developed foams were comprehensively evaluated. FTIR analysis confirmed the effective incorporation of the bio-polyol into the polyurethane matrix, showing a marked reduction in free isocyanate (–NCO) peaks and strengthened O–H/N–H hydrogen bonding interactions with increasing bio-polyol content. SEM and quantitative image analysis (ImageJ) demonstrated that the incorporation of the bio-polyol refined the average cell size and yielded a more homogeneous structure while maintaining an open-cell morphology in the range of 84.7–87.1%. This morphological development was supported by apparent density measurements, which slightly increased from 0.0835 g/cm^3^ for the Standard PU foam to 0.0852 g/cm^3^ for the 50 Bio PU foam and then decreased to 0.0703 and 0.0657 g/cm^3^ for the 75 Bio PU and 100 Bio PU foams, respectively. Accounting for density variances among samples, water absorption capacity was evaluated on both a gravimetric (%) and volumetric (g/cm^3^) basis. Despite the decrease in apparent density, up to a 22.48% reduction in gravimetric water absorption was recorded, which was attributed to increased capillary resistance from smaller cell dimensions and reduced free volume induced by intensified intermolecular hydrogen bonding. Despite the lower apparent density, a significant enhancement in the mechanical performance of the foams was achieved. The 75 Bio PU formulation (NCO/OH ≈ 1.80) exhibited the highest compression modulus (≈430 kPa vs. ≈120 kPa for the Standard PU foam), collapse stress (≈26 vs. ≈8 kPa) and compressive strength (≈30 vs. ≈8 kPa at 10% deformation according to ISO 844, and ≈175 vs. ≈40 kPa at 70% strain). Since the 75 Bio PU foam was ≈16% less dense than the Standard PU foam, its ≈5-fold higher density-normalised modulus demonstrated that this improvement originated from the bio-polyol-modified network and the refined cellular structure with the highest closed-cell fraction, rather than from density. In contrast, complete substitution (100 Bio PU, NCO/OH ≈ 1.83) resulted in the lowest modulus (≈90 kPa), owing to the loss of the polyether soft segment, an over-cross-linked network, thinner cell walls and the lowest density. These results confirm that the compressive behaviour is governed by the interplay between the NCO/OH index, polyol functionality, apparent density and cell morphology. Thermal conductivity measurements conducted at 100 °C to evaluate insulation stability under elevated operational temperatures (such as building roof/facade thermal loads and industrial hot pipeline insulation) revealed that the bio-based foams-maintained insulation efficiency (0.046–0.047 W m ^−1^ K ^−1^) closely comparable to the Standard PU foam (0.044 W m ^−1^ K ^−1^). Quantitative TGA/DTG analysis indicated that the degradation mechanism shifted upon incorporating the bio-polyol. The early-stage weight loss (T__%5_ and T__%15_) was ascribed to the low-temperature decomposition of lower-molecular-weight fractions and unreacted/labile linkages not fully integrated into the network. Conversely, in the main polymer network degradation, the 75 Bio PU foam exhibited superior thermal resistance and carbonization tendency, reaching a higher maximum degradation temperature (T __max%_ = 408 °C) and the highest char residue (15.68% at 600 °C) compared to the Standard PU foam (T __max%_ = 397 °C, char 11.00%). In UL-94 vertical burning tests, the absence of self-extinguishing behaviour across all samples indicated the need for supplementary flame-retardant additive systems. In conclusion, the CH/GL/PEG-400 bio-based polyol was proven to be a sustainable alternative in the production of semi-rigid polyurethane foams. Balancing low apparent density, high compression modulus and compressive strength, improved water resistance, maintained thermal insulation at 100 °C, and high char yield, the formulation with 75% bio-polyol substitution (75 Bio PU) emerges as the most balanced system. These bio-based foams demonstrate significant potential for application in building insulation, lightweight engineering systems, and auxiliary comfort components in the automotive interior industry.

## Figures and Tables

**Table 1:** Formulations for the preparation of standard PU foam and bio-based PU foam.

Sample	Polyether Polyol (g)	Bio-Based Polyol (g)	Isocyanate (g)	DBTDL (g)	H_2_O (g)	Surfactant (g)
Standard PU Foam	64	00	36	0.6	0.8	0.3
50 BİO PU Foam	32	32	36	0.6	0.8	0.3
75 BİO PU Foam	15	45	39	0.6	0.7	0.3
100 BİO PU Foam	00	60	39	0.6	0.6	0.3

**Table 2:** Foaming properties of standard and bio-based polyurethane foams.

Sample	Cream Time (s)	Free Rise Time (s)	Gel Time (s)	NCO/OH Index
Standard PU Foam	≈35	≈83	≈189	≈1.75
50 Bio PU Foam	≈34	≈85	≈190	≈1.58
75 Bio PU Foam	≈37	≈83	≈185	≈1.80
100 Bio PU Foam	≈40	≈81	≈187	≈1.83

**Table 3:** Calculated MALDI-TOF MS mass distribution and polydispersity data for chitosan.

*m*/*z* (*Mi*)	Signal Intensity (*Ni*)	Mn (Da) $\left(\frac{\Sigma \mathrm{NiMi}}{\Sigma \mathrm{Ni}}\right)$	Mw (Da) $\left(\frac{\Sigma {\mathrm{NiMi}}^{2}}{\Sigma \mathrm{NiMi}}\right)$	Dispersity Indices (Đ) $\left(\frac{\mathrm{Mw}}{\mathrm{Mn}}\right)$
23,123.248	594	34,260.2	42,336.5	1.23
26,871.271	263			
27,174.104	153			
59,308.616	183			
66,483.485	157			
147,257.215	143	162,126.1	162,482.0	1.002
161,015.449	1056			
161,031.392	519			
161,034.352	520			
161,077.605	191			
161,133.499	141			
188,218.813	183			
322,578.521	798	322,619.7	322,641.8	1.00
322,596.474	650			
322,612.274	667			
322,664.503	251			
322,680.362	234			
322,742.260	226			
322,824.931	265			
322,845.839	232			
322,937.294	284			
322,956.166	304			
322,967.651	285			
322,988.144	236			
323,078.090	340			
323,093.247	375			
323,512.807	160			
323,523.173	162			

**Table 4:** TGA/DTG results of Standard PU, 50 Bio PU, 75 Bio PU, and 100 Bio PU Foams.

	T_%5_	T_%10_	T_%15_	T_%40_	T_%max_	Char Residue (%)
Standard PU Foam	293	325	350	387	397	11.0
50 BİO PU Foam	129	163	182	315	375	12.86
75 BİO PU Foam	129	183	213	321	408	15.68
100 BİO PU Foam	149	183	196	272	388	8.17

**Table 5:** Quantitative compressive parameters of the Standard PU, 50 Bio PU, 75 Bio PU and 100 Bio PU foams in relation to their NCO/OH index, apparent density and open-cell content.

Parameter	Standard PU Foam	50 Bio PU Foam	75 Bio PU Foam	100 Bio PU Foam
NCO/OH index	1.75	1.58	1.80	1.83
Apparent density, ρ (g/cm^3^)	0.0835 ± 0.0012	0.0852 ± 0.0082	0.0703 ± 0.0097	0.0657 ± 0.0097
Open-cell content (%)	86.7	87.1	84.7	86.2
Compression modulus, E (kPa)	≈120	≈120	≈430	≈90
ε_c_ (%)/σ_c_ (kPa)	≈7/≈8	≈14/≈15	≈8/≈26	≈10/≈8
Compressive strength, σ_10_ (kPa) (ISO 844)	≈8	≈11	≈30	≈8
σ_50_ (kPa)	≈17	≈42	≈69	≈34
σ_70_ (kPa, end of test)	≈40	≈115	≈175	≈104

## Data Availability

The datasets presented in this article are not readily available because the data are part of an ongoing study. Requests to access the datasets should be directed to the Corresponding Author.

## Supplementary Materials

The following supporting information can be downloaded at: https://www.mdpi.com/article/10.3390/polym18212770/s1: Table S1: Supplementary data related to the characterization of polyurethane foam samples.

## Abbreviations

The following abbreviations are used in this manuscript:

CH	Chitosan
GL	Glycerol
PEG 400	Polyethylene glycol 400
PU	Polyurethane
TGA	Thermogravimetric Analysis
FTIR	Fourier-Transform Infrared Spectroscopy
DTG	Derivative Thermogravimetry
SEM	Scanning Electron Microscopy
DBTDL	Dibutyltin Dilaurate
PMDI	Polymeric Methylene Diphenyl Diisocyanate
EC	Ethylene Carbonate
Glycidol	Glycidol
MALDI-TOF MS	Matrix-Assisted Laser Desorption/Ionization Time-of-Flight Mass Spectrometry
–NCO	Isocyanate Group
