# Supplementary material for: Synthesis of Chitosan-Based Bio-Polyols Using Glycerol and PEG-400 for Sustainable Semi-Rigid Polyurethane Foams
Source: Polymers (Basel). 2026 Nov 6;18(21):2770. doi: 10.3390/polym18212770 (PMC13766078; doi:10.3390/polym18212770)
Supplement: Supplementary file 1 [file polymers-18-02770-s001.zip › polymers-4627688-supplementary.pdf]

SYNTHESIS OF CHITOSAN-BASED BIO-POLYOLS USING GLYCEROL AND PEG-400 FOR SUSTAINABLE SEMI-RIGID POLYURETHANE FOAMS

MALDI-TOF MS Analysis Results and Molecular Weight Distribution Calculation Report

Table S1. MALDI-TOF Analysis Result: Peak Values, Signal Intensities, and Calculated Molecular Weight Parameters (M_n, M_w, Đ) for Chitosan-Based Bio-Polyols

m/z (M_i)	Signal Intensity (N_i)	M_n (Da) ($\Sigma N_i M_i / \Sigma N_i$)	M_w (Da) ($\Sigma N_i M_i^2 / \Sigma N_i M_i$)	Dispersity Index (Đ) (M_w / M_n)
15678.854	339	58,427.7	73,048.0	1.250
29621.123	320			
29791.285	376			
30038.273	321			
30642.025	406			
30647.300	389			
30917.681	302			
30923.626	327			
30991.662	292			
31048.860	326			
32743.904	399			
32749.689	317			
33329.071	437			
33331.315	433			
33903.079	631			
33931.911	318			
34395.119	442			
35916.787	353			
35938.026	351			
35964.474	386			
36161.257	320			
37756.369	351			
37883.325	293			
38268.763	292			
38347.595	324			
38926.678	293			
38926.678	294			
38960.338	1154			

m/z (M_i)	Signal Intensity (N_i)	M_n (Da) ($\Sigma N_i M_i / \Sigma N_i$)	M_w (Da) ($\Sigma N_i M_i^2 / \Sigma N_i M_i$)	Dispersity Index (Đ) (M_w / M_n)
38982.478	321			
39859.004	323			
41004.540	364			
42076.215	293			
42887.478	293			
42969.265	340			
42973.762	314			
44753.983	445			
46974.246	635			
46977.284	659			
47002.251	324			
49028.264	296			
51075.343	298			
52980.872	355			
52989.050	346			
58382.908	320			
59022.827	315			
61825.996	372			
64899.484	786			
66127.330	337			
70084.497	1855			
75154.522	360			
77590.302	309			
79511.384	332			
96498.036	1798			
100155.589	296			
100936.216	309			
101577.146	421			
103822.950	310			
118690.131	1411			
118706.033	687			
118723.319	570			
192389.468	1653	207,066.7	207,233.2	1.001
192427.418	294			

m/z (M_i)	Signal Intensity (N_i)	M_n (Da) ($\Sigma N_i M_i / \Sigma N_i$)	M_w (Da) ($\Sigma N_i M_i^2 / \Sigma N_i M_i$)	Dispersity Index (Đ) (M_w / M_n)
192444.629	379			
200330.689	1612			
207824.792	2993			
207846.647	2410			
207867.430	2592			
207940.070	701			
207953.826	677			
208001.885	769			
208070.737	316			
208079.595	352			
208334.001	311			
211849.160	2936			
211912.596	778			
211931.199	1122			
211948.961	1008			
212029.042	643			
212074.343	293			
215551.937	341			
299182.646	3132	317,004.1	318,352.9	1.004
310160.791	2104			
310231.539	940			
310287.556	572			
310315.956	438			
310336.715	398			
310381.321	482			
310398.226	553			
310470.996	407			
310487.284	405			
310521.225	428			
310645.443	295			
310664.829	313			
310718.809	385			
310796.126	376			
338117.698	2718			

m/z (M_i)	Signal Intensity (N_i)	M_n (Da) ($\Sigma N_i M_i / \Sigma N_i$)	M_w (Da) ($\Sigma N_i M_i^2 / \Sigma N_i M_i$)	Dispersity Index (Đ) (M_w / M_n)
338346.216	300			
338643.629	306			
338863.918	312			
425056.932	299			

Calculation Formulas and Definitions:

- **M_n (Number-Average Molecular Weight):** $M_n = \Sigma(N_i \cdot M_i) / \Sigma N_i$
- **M_w (Weight-Average Molecular Weight):** $M_w = \Sigma(N_i \cdot M_i^2) / \Sigma(N_i \cdot M_i)$
- **Dispersity Index (Đ):** $Đ = M_w / M_n$
- *Note: All 100 peak data points from the provided MALDI-TOF MS spectra have been retained without omitting any values.*
